# Mishel theory-based perioperative nursing after trabeculectomy for primary glaucoma: effects on illness uncertainty, self-management, and quality of life

**DOI:** 10.3389/fpsyt.2026.1884293

**Published:** 2026-10-14

**Authors:** Qi-Ying Ding, Chun Li, Xue-Man Yin

**Affiliations:** Ophthalmology, The First Affiliated Hospital of Suzhou University, Suzhou, Jiangsu, China

**Keywords:** illness uncertainty, perioperative nursing, primary glaucoma, self-management, trabeculectomy

## Abstract

**Objective:**

To evaluate the effects of a perioperative nursing intervention based on Mishel’s Uncertainty in Illness Theory in patients with primary glaucoma undergoing trabeculectomy.

**Methods:**

This single-center, two-arm, parallel-group randomized controlled study enrolled 198 patients undergoing first trabeculectomy at The First Affiliated Hospital of Suzhou University between February 2024 and December 2025. Participants were randomly allocated 1:1 to routine perioperative ophthalmic nursing or routine care plus a structured Mishel theory-based nursing intervention. Assessments were conducted 1 day before surgery (T0), at discharge (T1), and at 1 month (T2) and 3 months (T3) after surgery. The primary outcome was illness uncertainty measured using the Mishel Uncertainty in Illness Scale. Secondary outcomes included psychological status, discharge readiness, self-management, medication adherence, sleep quality, vision-related quality of life, satisfaction with nursing care, postoperative ophthalmic outcomes, and complications. All randomized participants completed follow-up and were analyzed according to their assigned groups.

**Results:**

After Holm adjustment, the intervention group had lower illness-uncertainty scores at T1, T2, and T3; lower anxiety scores at T1 and T3 and lower depression scores at T2 and T3; higher discharge-readiness and satisfaction-with-nursing-care at T1; higher self-management and medication-adherence scores and lower sleep-quality scores at T2 and T3; and lower GQL-15 scores at T3. At T3, the model-estimated between-group differences were -8.13 points for illness uncertainty (95% CI, -10.74 to -5.53), 5.64 points for self-management (95% CI, 4.08-7.19), 6.05 points for medication adherence (95% CI, 2.98-9.12), and -3.71 points for GQL-15 (95% CI, -5.41 to -2.00). At discharge, the between-group difference in discharge readiness was 1.23 points (95% CI, 0.99-1.47). No statistically significant between-group differences were detected in postoperative intraocular-pressure trajectories, visual-acuity recovery, medication-free status, postoperative procedures, or complications within 3 months.

**Conclusion:**

In this single-center randomized study, Mishel theory-based perioperative nursing was associated with greater improvements in illness uncertainty and several psychological, behavioral, and recovery-related patient-reported outcomes after trabeculectomy. No statistically significant differences were detected in short-term objective ophthalmic outcomes or postoperative complications.

## Introduction

Glaucoma is a leading cause of irreversible visual impairment and blindness worldwide, and the number of affected individuals aged 40–80 years is projected to reach approximately 111.8 million by 2040 (1, 2). Asia bears a particularly substantial share of this burden, and primary angle-closure glaucoma (PACG) is considerably more prevalent in Asian populations than in European populations (3, 4). Elevated intraocular pressure (IOP) remains the principal modifiable risk factor for glaucomatous optic nerve damage and progressive visual-field loss (1). Glaucoma management generally follows a stepwise approach involving topical medications, laser treatment, and surgery when target IOP cannot be achieved or structural or functional progression persists (1, 3).

Trabeculectomy remains a standard subconjunctival filtration procedure for achieving substantial and sustained IOP reduction by creating an alternative aqueous-humor outflow pathway from the anterior chamber to the subconjunctival space (5, 6). Despite its effectiveness, glaucoma and its treatment may impose considerable functional and psychosocial burdens, including impaired daily activities, reduced independence, treatment burden, and poorer vision-related quality of life (7). Patients undergoing trabeculectomy must also cope with concerns regarding surgical risk, postoperative symptoms, filtering-bleb function, complications, recovery, and long-term IOP control.

In addition to visual impairment, patients with glaucoma frequently experience anxiety, depression, and uncertainty regarding disease progression and future vision (8, 9). Illness uncertainty refers to an inability to assign clear meaning to illness-related events or predict their consequences because available information is ambiguous, complex, inconsistent, or insufficient (10, 11). The chronic, progressive, and sometimes asymptomatic nature of glaucoma, together with complex long-term treatment and fear of irreversible visual loss, may increase this uncertainty. Evidence from surgical populations similarly indicates that greater illness uncertainty is associated with preoperative anxiety, poorer psychological adjustment, and less effective self-care and adaptation (12–14).

Mishel’s Uncertainty in Illness Theory provides a structured framework for explaining how patients perceive, interpret, appraise, and respond to illness-related uncertainty (11, 15, 16). Unlike routine health education, which primarily delivers standardized disease and treatment information, a Mishel theory-based intervention identifies individual sources of ambiguity, adapts communication to patients’ cognitive capacities, strengthens professional and family support as structure providers, and promotes adaptive appraisal and coping. From a psychosomatic perspective, metacognitive capacity may also influence how patients interpret illness-related information and translate it into emotional and behavioral responses, although supporting evidence has largely been derived from psychiatric populations (17). In glaucoma surgery, uncertainty may therefore function not only as an emotional response but also as a behavioral determinant influencing symptom interpretation, medication adherence, help-seeking, follow-up participation, self-management, and perceived recovery. The major theoretical constructs and their corresponding nursing strategies are summarized in **Table 1**.

**Table 1:** Perioperative nursing intervention based on Mishel’s Uncertainty in Illness Theory.

Theoretical component	Preoperative intervention (T0)	Intraoperative intervention	Postoperative intervention (T1, T2, T3)
Stimuli frame	1) Assess understanding of glaucoma, trabeculectomy, and postoperative recovery. 2) Explain the disease, surgical purpose, procedure, common reactions, and follow-up requirements.	1) Re-explain anesthesia and intraoperative cooperation. 2) Briefly explain key steps and possible transient discomfort.	1) Explain common postoperative symptoms and bleb-related observations. 2) Reinforce recovery progress, IOP fluctuation, and common postoperative issues.
Cognitive capacities	1) Provide individualized education according to age, education, visual function, and comprehension. 2) Use simple verbal explanation, written materials, and teach-back.	1) Give brief and clear instructions during the procedure.	1) Reassess understanding of eye-drop use, activity restrictions, warning signs, and follow-up. 2) Reinforce insufficiently understood information.
Structure providers	1) Establish a physician-nurse-patient-family support system. 2) Encourage caregiver participation.	1) Maintain consistent communication. 2) Provide reassurance when needed.	1) Provide discharge guidance. 2) Conduct follow-up by telephone and encourage family support with medication use and follow-up visits.
Appraisal	1) Identify concerns about blindness, surgical failure, IOP control, and prognosis. 2) Help patients view surgery as a positive treatment.	1) Provide immediate explanation and reassurance for anxiety and fear.	1) Correct unrealistic expectations about recovery and treatment effect. 2) Help patients view recovery uncertainty as manageable.
Coping strategies	1) Encourage active coping. 2) Teach breathing relaxation and emotional regulation.	1) Guide stable positioning and cooperation. 2) Use supportive language to maintain calmness.	1) Instruct patients to use eye drops on time, avoid rubbing the eye, bending, and straining, and protect the filtering bleb. 2) Reinforce medication adherence, follow-up planning, and emotional regulation.
Adaptation	1) Strengthen basic disease understanding and surgical confidence.	1) Support smooth intraoperative cooperation and emotional stability.	1) Support early self-management after discharge. 2) Reinforce adaptation in disease understanding, emotional status, and daily management during follow-up.

Delivery schedule: Five scheduled contacts were provided at T0, during surgery, at discharge (T1), and by telephone at T2 and T3.

Evidence on theory-based perioperative nursing specifically designed for patients with primary glaucoma undergoing trabeculectomy remains limited. Therefore, this study aimed to evaluate whether a structured perioperative nursing intervention based on Mishel’s Uncertainty in Illness Theory was associated with improvements in illness uncertainty, psychological adjustment, discharge readiness, self-management, medication adherence, sleep quality, and vision-related quality of life in this population.

## Materials and methods

### Ethics statement

This study was approved by the Ethics Committee of The First Affiliated Hospital of Suzhou University. Written informed consent was obtained from all participants. The study was conducted in accordance with the Declaration of Helsinki.

### Sample size calculation

The sample size was calculated using the Mishel Uncertainty in Illness Scale (MUIS) score as the primary outcome. Based on an estimated standard deviation of 11.5 points in patients undergoing glaucoma surgery (9), an expected between-group difference of 5 points, a two-sided α of 0.05, 80% power, and 1:1 allocation, 84 participants were required per group. Allowing for 15% attrition, the target sample size was 99 participants per group, for a total of 198.

### Study setting and participants

This single-center, two-arm, parallel-group randomized controlled trial was conducted in the Department of Ophthalmology of The First Affiliated Hospital of Suzhou University. The department serves as a regional referral center for glaucoma and performs approximately 150 trabeculectomy procedures annually. Between February 2024 and December 2025, 258 patients were screened and 198 eligible participants were enrolled and randomly allocated 1:1 to the control or intervention group (**Figure 1**). Eligible participants were aged ≥18 years, had primary glaucoma requiring first trabeculectomy because of inadequate IOP control despite maximally tolerated medical and/or laser treatment or documented structural or functional progression, were able to complete the questionnaires and follow-up, and provided written informed consent. Exclusion criteria were secondary glaucoma; previous incisional glaucoma surgery or conjunctiva-related ocular surgery likely to affect filtration outcomes; active ocular infection or inflammation, corneal opacity, vitreous in the anterior chamber, or another ocular condition interfering with evaluation; severe neurological or systemic disease affecting visual assessment; pregnancy or breastfeeding; or inability to complete follow-up. Eligibility was confirmed by an ophthalmologist, participants were enrolled by a research nurse, and allocation was implemented after baseline assessment. The diagnoses of POAG and PACG were made according to the Asia-Pacific Glaucoma Guidelines (3), based on gonioscopic findings, glaucomatous optic neuropathy, and corresponding visual-field loss.

**Figure 1:**
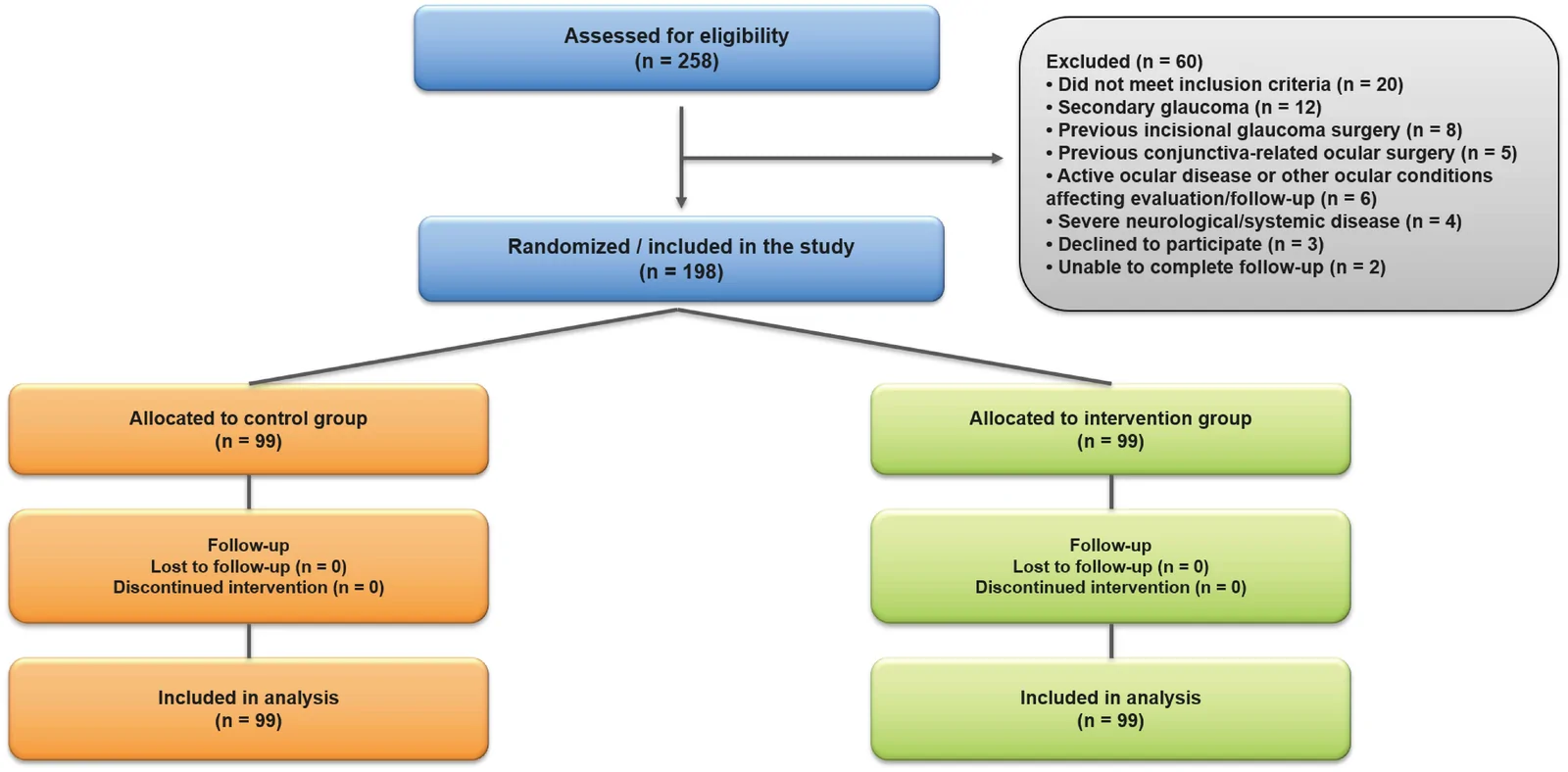
CONSORT flow diagram of patient recruitment, screening, and group allocation.

### Randomization and blinding

An independent statistician generated the random sequence using SPSS version 22.0 and was not involved in recruitment or intervention delivery. Allocations were placed in sequentially numbered, opaque, sealed envelopes and disclosed only after enrollment and baseline assessment. Participants and intervention nurses could not be blinded because of the nature of the intervention, and outcome assessors and statistical analysts were not formally blinded. Reporting followed the CONSORT statement, the TIDieR checklist, and the CONSORT extension for nonpharmacologic trials (18–20).

### Surgical and postoperative medical protocol

All trabeculectomies were performed by experienced glaucoma surgeons from the same departmental surgical team following a standardized institutional protocol. A superior conjunctival flap and a partial-thickness scleral flap were created, followed by sclerostomy and peripheral iridectomy. The scleral flap was secured with sutures, and the conjunctiva was closed in a watertight manner. Intraoperative antimetabolite use was determined according to the patient’s scarring risk and the institutional protocol. Both groups received the same postoperative medical management, including topical corticosteroid and antibiotic eye drops and adjustment of preoperative glaucoma medications according to IOP and filtering-bleb function. Scheduled ophthalmic follow-up was conducted during the early postoperative period and at 1 and 3 months. Visual acuity, IOP, anterior-chamber depth, ocular inflammation, filtering-bleb morphology, wound leakage, and complications were evaluated at each visit. Laser suture lysis, releasable-suture removal, subconjunctival antifibrotic injection, or bleb needling was performed when clinically indicated. All surgical and postoperative medical decisions were made by the treating ophthalmologists independently of nursing-group allocation.

### Intervention

The control group received routine standardized ophthalmic nursing delivered by ward ophthalmic nurses according to the institutional trabeculectomy care pathway and relevant clinical recommendations (1, 21, 22). Routine care included preoperative verification and assessment, general education about surgery and anesthesia, intraoperative safety preparation and cooperation guidance, postoperative observation, eye protection, prescribed medication administration, eye-drop instruction, written discharge guidance, activity restrictions, warning signs, and routine ophthalmic follow-up. The control group did not receive structured uncertainty assessment, teach-back reinforcement, individualized appraisal and coping guidance, or scheduled nursing telephone follow-up at T2 and T3.

Patients in the intervention group received routine care plus a structured Mishel theory-based nursing program (**Table 1**). For this study, the intervention was conceptualized as a perioperative-to-post-discharge nursing pathway that began before surgery, continued through the intraoperative and discharge phases, and extended to telephone follow-up at 1 and 3 months. All five intervention contacts were delivered by a designated team of ophthalmic nurses from the same department; all had at least 5 years of ophthalmic clinical experience and received standardized training in Mishel’s theory, the intervention protocol, teach-back communication, and postoperative glaucoma management. Thus, although contacts occurred in different care settings, the intervention was delivered within one ophthalmic nursing team rather than by nurses from different specialties. The program addressed six components: stimuli frame, cognitive capacities, structure providers, appraisal, coping, and adaptation (10, 23). Before surgery, nurses assessed patients’ understanding and concerns and provided individualized education using verbal explanations, written materials, and teach-back. During surgery, a trained intervention nurse provided brief cooperation guidance and reassurance in coordination with the surgical team. At discharge and during telephone follow-up, nurses reinforced postoperative symptoms, eye-drop use, activity restrictions, warning signs, emotional regulation, follow-up attendance, and self-management. Core content was standardized, while communication and reinforcement were individualized according to age, education, visual function, comprehension, and identified concerns. Family participation was encouraged when appropriates.

### Outcome measurements

Assessments were conducted 1 day before surgery (T0), at discharge (T1), and at 1 month (T2) and 3 months (T3) after surgery. The primary outcome was illness uncertainty, assessed using the 28-item adult Mishel Uncertainty in Illness Scale (MUIS), which evaluates ambiguity, complexity, inconsistency, and unpredictability on a 5-point Likert scale and yields a total score of 28–140, with higher scores indicating greater uncertainty (9, 10). Secondary patient-reported outcomes included anxiety and depression, assessed using the 7-item HADS-A and HADS-D subscales (range, 0–21) (24); discharge readiness, assessed using the 12-item Readiness for Hospital Discharge Scale (RHDS), with higher mean item scores indicating greater readiness (25); self-management, assessed using the 17-item Glaucoma Self-Management Questionnaire (GSMQ; range, 17–68) (26); medication adherence, assessed using the 27-item Glaucoma Treatment Compliance Assessment Tool (GTCAT-S) (27); sleep quality, assessed using the Pittsburgh Sleep Quality Index (PSQI; range, 0–21), with higher scores indicating poorer sleep quality (28); vision-related quality of life, assessed using the Chinese version of the 15-item Glaucoma Quality of Life questionnaire (GQL-15-CHI), with higher scores indicating poorer vision-related quality of life (29); and satisfaction with nursing care, assessed using the 19-item satisfaction subscale of the Newcastle Satisfaction with Nursing Scales (NSNS), with scores converted to a 0–100 scale (30). The psychometric properties of these instruments are summarized in **Supplementary Table S2**.

MUIS, HADS-A, and HADS-D were assessed at T0–T3; RHDS-SF and NSNS at T1; and GSMQ, GTCAT-S, PSQI, and GQL-15 at T0, T2, and T3. Objective ophthalmic outcomes included postoperative IOP, logMAR best-corrected visual acuity, glaucoma-medication burden, and postoperative procedures. Postoperative complications within 3 months were identified by the treating ophthalmologist from scheduled examinations and medical records using study-specific criteria informed by previous trabeculectomy studies (31–33). Transient hypotony was defined as IOP ≤6 mmHg with resolution within 3 months; wound or bleb leakage as a positive Seidel test; shallow anterior chamber as documented shallowing requiring treatment; choroidal detachment or effusion as confirmed by fundus examination or B-scan ultrasonography; hyphema as visible anterior-chamber blood; and transient IOP spike as IOP ≥30 mmHg or an elevation requiring additional pressure-lowering treatment within the first postoperative month. A patient could be counted in more than one complication category, whereas composite outcomes counted each patient once. Maximum severity was retrospectively classified according to the most intensive management required: mild events required observation or topical treatment, moderate events required additional medication or an office-based procedure, and severe events required anterior-chamber reformation, wound or bleb revision, or reoperation.

### Statistical analysis

Data were analyzed using SPSS version 26.0, and GraphPad Prism was used for figure preparation. Continuous variables are presented as mean ± SD or median (IQR), and categorical variables as n (%). Baseline comparisons used independent-samples t tests, Mann–Whitney U tests, chi-square tests, or Fisher’s exact tests, as appropriate. Model assumptions were assessed using Q-Q and residual-versus-fitted plots. Repeated patient-reported outcomes were analyzed using linear mixed-effects models with group, categorical time, and group × time interaction as fixed effects and a participant-specific random intercept with a compound-symmetric covariance structure. RHDS-SF and NSNS were compared using independent-samples t tests. Postoperative IOP and logMAR BCVA were analyzed using generalized estimating equations with an exchangeable correlation structure and adjustment for the corresponding baseline value; time-specific differences were estimated using baseline-adjusted linear models. Medication counts and maximum complication severity were compared using Mann–Whitney U tests. Binary outcomes were compared using chi-square or Fisher’s exact tests, with risk ratios and 95% CIs reported where appropriate. No data were missing. Holm adjustment was applied to 19 postbaseline patient-reported outcome contrasts, whereas nominal *P* values were reported for exploratory ophthalmic, complication, procedure, and HADS-threshold analyses. Two-sided *P* values <0.05 were considered statistically significant.

## Results

### Baseline demographic and clinical characteristics

There were no statistically significant differences in baseline demographic or clinical characteristics between the two groups (all *P >* 0.05, **Table 2**). The mean age was 66.87 ± 7.26 years in the control group and 68.10 ± 7.55 years in the intervention group (*P =* 0.245). Baseline intraocular pressure was 24.60 mmHg (IQR, 22.85-27.15) in the control group and 25.90 mmHg (IQR, 22.40-28.15) in the intervention group (*P =* 0.322). The median number of baseline glaucoma medications was 3.00 (IQR, 2.00-3.00) in both groups (*P =* 0.582). Baseline best-corrected visual acuity, central corneal thickness, axial length, cup-to-disc ratio, and visual-field mean deviation were also comparable between groups. The distributions of sex, education level, glaucoma diagnosis, study eye, lens status, hypertension, and diabetes were similarly balanced between the two groups (all *P >* 0.05), indicating good baseline comparability.

**Table 2:** Baseline demographic and clinical characteristics of the two groups.

Characteristic	Control group (n = 99)	Intervention group (n = 99)	*P*
Age, years	66.87 ± 7.26	68.10 ± 7.55	0.245
Sex, n (%)			0.653
Male	32 (32.3)	36 (36.4)	
Female	67 (67.7)	63 (63.6)	
Education level			0.434
Primary school or below	41 (41.4)	46 (46.5)	
Junior high school	44 (44.4)	38 (38.4)	
Senior high school	9 (9.1)	13 (13.1)	
College or above	5 (5.1)	2 (2.0)	
Hypertension, n (%)			0.116
Yes	38 (38.4)	50 (50.5)	
No	61 (61.6)	49 (49.5)	
Diabetes, n (%)			0.421
Yes	12 (12.1)	17 (17.2)	
No	87 (87.9)	82 (82.8)	
Diagnosis, n (%)			0.556
POAG	39 (39.4)	34 (34.3)	
PACG	60 (60.6)	65 (65.7)	
Study eye, n (%)			0.775
OD	52 (52.5)	55 (55.6)	
OS	47 (47.5)	44 (44.4)	
Lens status, n (%)			0.776
Phakic	50 (50.5)	53 (53.5)	
Pseudophakic	49 (49.5)	46 (46.5)	
Baseline IOP, mmHg	24.60 (22.85-27.15)	25.90 (22.40-28.15)	0.322
Baseline medications, n	3.00 (2.00-3.00)	3.00 (2.00-3.00)	0.582
BCVA, decimal	0.51 ± 0.18	0.55 ± 0.17	0.157
Central corneal thickness, μm	525.28 ± 12.56	524.27 ± 12.94	0.578
Axial length, mm	22.70 (22.20-23.26)	22.91 (22.26-23.61)	0.141
Cup-to-disc ratio	0.77 ± 0.09	0.75 ± 0.09	0.277
Visual field MD, dB	-13.36 (-16.66--10.15)	-12.89 (-15.18--9.86)	0.318

Continuous variables are presented as mean ± SD or median (IQR), as appropriate, and categorical variables are presented as n (%). POAG, primary open-angle glaucoma; PACG, primary angle-closure glaucoma; OD, right eye; OS, left eye; IOP, intraocular pressure; BCVA, best-corrected visual acuity; CCT, central corneal thickness; MD, mean deviation.

### Longitudinal patient-reported outcomes

Linear mixed-effects models demonstrated significant time effects and group × time interactions for MUIS, HADS-A, and HADS-D scores (all *P <* 0.001; **Figure 2**; **Table 3**). Significant time effects and group × time interactions were also observed for GSMQ, GTCAT-S, PSQI, and GQL-15 scores (all *P <* 0.001; **Figure 3**; **Table 3**). The intervention group showed greater reductions in illness uncertainty during follow-up. The model-estimated between-group differences in MUIS scores were -5.58 points at discharge (95% CI, -8.18 to -2.97), -7.84 points at 1 month (95% CI, -10.44 to -5.23), and -8.13 points at 3 months (95% CI, -10.74 to -5.53; all Holm-adjusted *P <* 0.001). HADS-A scores were lower in the intervention group at discharge and 3 months after Holm adjustment; the 1-month comparison did not remain statistically significant (adjusted *P =* 0.053). HADS-D scores were lower at 1 and 3 months, whereas the discharge comparison was attenuated after adjustment (adjusted *P =* 0.053). At T3, HADS-A scores ≥8 occurred in 26 patients (26.3%) in the control group and 13 (13.1%) in the intervention group (Fisher’s exact *P =* 0.031), while HADS-D scores ≥8 occurred in 35 (35.4%) and 13 (13.1%) patients, respectively (*P <* 0.001). The intervention group also demonstrated better self-management behavior and medication adherence. At 3 months, the between-group differences were 5.64 points for GSMQ (95% CI, 4.08-7.19; adjusted *P <* 0.001) and 6.05 points for GTCAT-S (95% CI, 2.98-9.12; adjusted *P =* 0.001). PSQI scores were lower in the intervention group at 1 and 3 months. The 1-month GQL-15 difference did not remain significant after Holm adjustment (adjusted *P =* 0.053), whereas the 3-month difference remained significant (-3.71; 95% CI, -5.41 to -2.00; adjusted *P <* 0.001). At discharge, the intervention group had greater discharge readiness and satisfaction with nursing care, with between-group differences of 1.23 points for RHDS-SF (95% CI, 0.99-1.47) and 5.75 points for NSNS (95% CI, 3.87-7.64; both Holm-adjusted *P <* 0.001).

**Figure 2:**
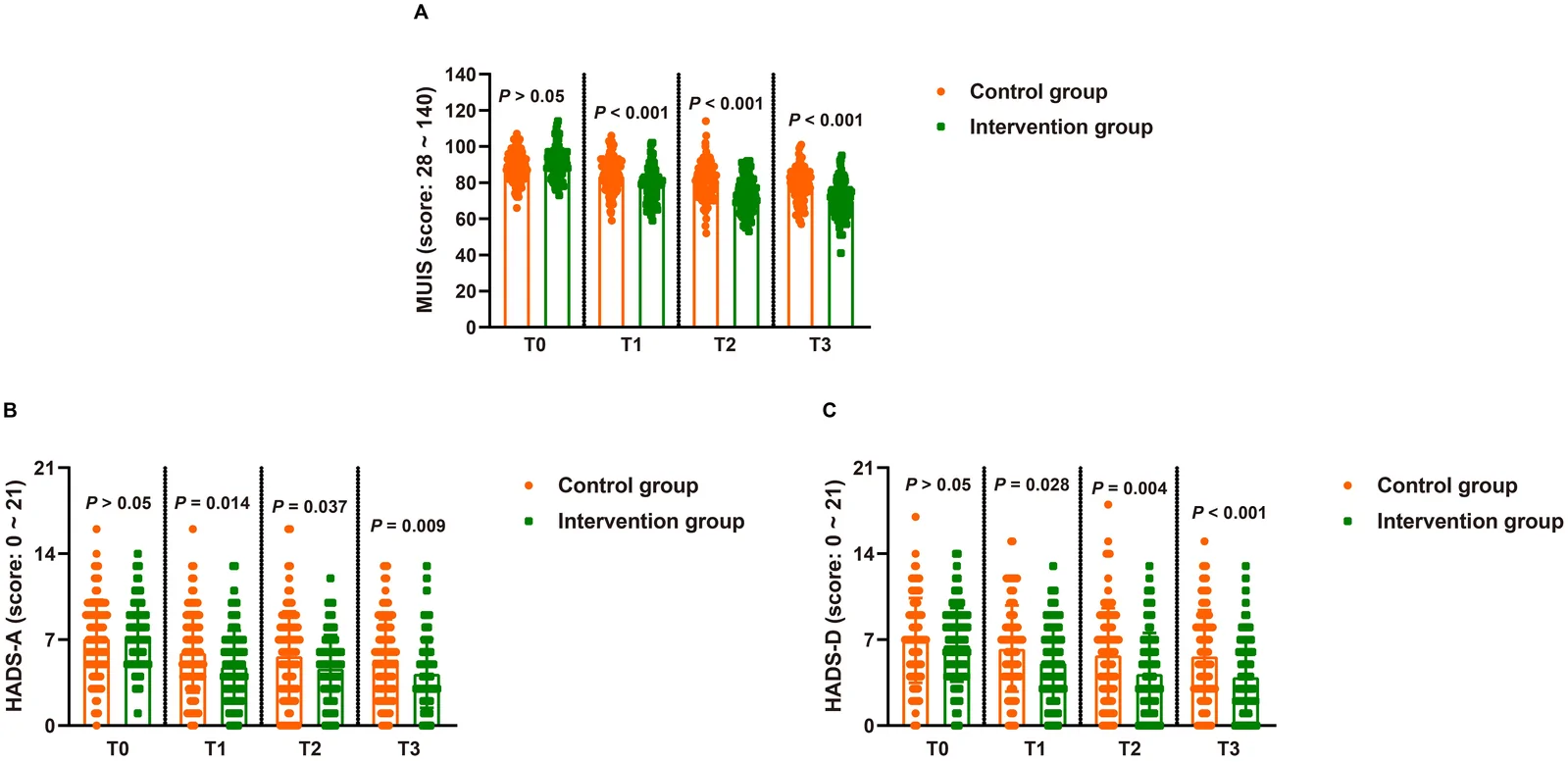
Comparisons of illness uncertainty and anxiety/depression scores between the two groups at different time points. **(A)** Mishel Uncertainty in Illness Scale (MUIS) scores, **(B)** Hospital Anxiety and Depression Scale-Anxiety subscale (HADS-A) scores, and **(C)** Hospital Anxiety and Depression Scale-Depression subscale (HADS-D) scores in the control group and intervention group at 1 day before surgery (T0), on the day of discharge (T1), 1 month after surgery (T2), and 3 months after surgery (T3). Values are presented as mean ± SD.

**Table 3:** Longitudinal patient-reported outcomes and model-based between-group differences.

Outcome	Control group (n = 99)	Intervention group (n = 99)	Mean difference (95% CI)	*P*	Holm-adjusted *P*
MUIS					
T0	89.07 ± 8.24	90.26 ± 8.56	1.19 (-1.41 to 3.80)	0.370	—
T1	83.94 ± 9.57	78.36 ± 9.63	-5.58 (-8.18 to -2.97)	**< 0.001**	**< 0.001**
T2	80.66 ± 10.07	72.82 ± 9.65	-7.84 (-10.44 to -5.23)	**< 0.001**	**< 0.001**
T3	78.65 ± 9.40	70.52 ± 9.93	-8.13 (-10.74 to -5.53)	**< 0.001**	**< 0.001**
HADS-A					
T0	7.13 ± 3.11	7.36 ± 2.54	0.23 (-0.61 to 1.07)	0.589	—
T1	5.96 ± 3.27	4.83 ± 2.91	-1.13 (-1.97 to -0.29)	**0.008**	**0.039**
T2	5.70 ± 3.60	4.69 ± 2.69	-1.01 (-1.85 to -0.17)	**0.019**	0.053
T3	5.42 ± 3.23	4.25 ± 2.80	-1.17 (-2.01 to -0.33)	**0.006**	**0.038**
HADS-D					
T0	6.94 ± 3.45	6.59 ± 3.00	-0.35 (-1.29 to 0.58)	0.459	—
T1	6.28 ± 3.50	5.15 ± 3.05	-1.13 (-2.07 to -0.20)	**0.018**	0.053
T2	5.77 ± 3.79	4.24 ± 3.30	-1.53 (-2.46 to -0.59)	**0.001**	**0.011**
T3	5.69 ± 3.73	3.98 ± 3.06	-1.71 (-2.64 to -0.77)	**< 0.001**	**0.003**
GSMQ					
T0	50.02 ± 5.32	50.02 ± 4.91	0.00 (-1.55 to 1.55)	1.000	—
T2	53.12 ± 6.30	57.27 ± 5.49	4.15 (2.60 to 5.70)	**< 0.001**	**< 0.001**
T3	53.26 ± 5.88	58.90 ± 5.57	5.64 (4.08 to 7.19)	**< 0.001**	**< 0.001**
GTCAT-S					
T0	103.80 ± 9.38	102.92 ± 9.56	-0.88 (-3.95 to 2.19)	0.575	—
T2	108.44 ± 11.51	113.82 ± 11.81	5.37 (2.30 to 8.44)	**< 0.001**	**0.005**
T3	109.57 ± 11.69	115.62 ± 12.16	6.05 (2.98 to 9.12)	**< 0.001**	**0.001**
PSQI					
T0	6.26 ± 1.90	6.28 ± 2.18	0.02 (-0.63 to 0.67)	0.951	—
T2	5.08 ± 2.48	4.20 ± 2.57	-0.88 (-1.53 to -0.23)	**0.008**	**0.039**
T3	5.00 ± 2.48	4.02 ± 2.33	-0.98 (-1.63 to -0.33)	**0.003**	**0.021**
GQL-15					
T0	28.06 ± 5.80	27.82 ± 5.78	-0.24 (-1.95 to 1.46)	0.781	—
T2	24.55 ± 6.52	22.63 ± 6.05	-1.92 (-3.63 to -0.21)	**0.027**	0.053
T3	24.63 ± 7.30	20.92 ± 5.28	-3.71 (-5.41 to -2.00)	**< 0.001**	**< 0.001**
RHDS-SF					
T1	7.17 ± 0.85	8.41 ± 0.85	1.23 (0.99 to 1.47)	**< 0.001**	**< 0.001**
NSNS					
T1	82.81 ± 6.88	88.56 ± 6.54	5.75 (3.87 to 7.64)	**< 0.001**	**< 0.001**

Values are mean ± SD. Mean differences are intervention minus control. Repeated patient-reported outcomes were analyzed using linear mixed-effects models; RHDS-SF and NSNS, assessed only at discharge, were compared using independent-samples t tests. Holm-adjusted P values account for 19 postbaseline contrasts. MUIS, Mishel Uncertainty in Illness Scale; HADS-A/HADS-D, Hospital Anxiety and Depression Scale anxiety/depression subscales; GSMQ, Glaucoma Self-Management Questionnaire; GTCAT-S, Glaucoma Treatment Compliance Assessment Tool-Short Form; PSQI, Pittsburgh Sleep Quality Index; GQL-15, Glaucoma Quality of Life-15; RHDS-SF, Readiness for Hospital Discharge Scale-Short Form; NSNS, Newcastle Satisfaction with Nursing Scales. Statistically significant *P* values are shown in bold.

**Figure 3:**
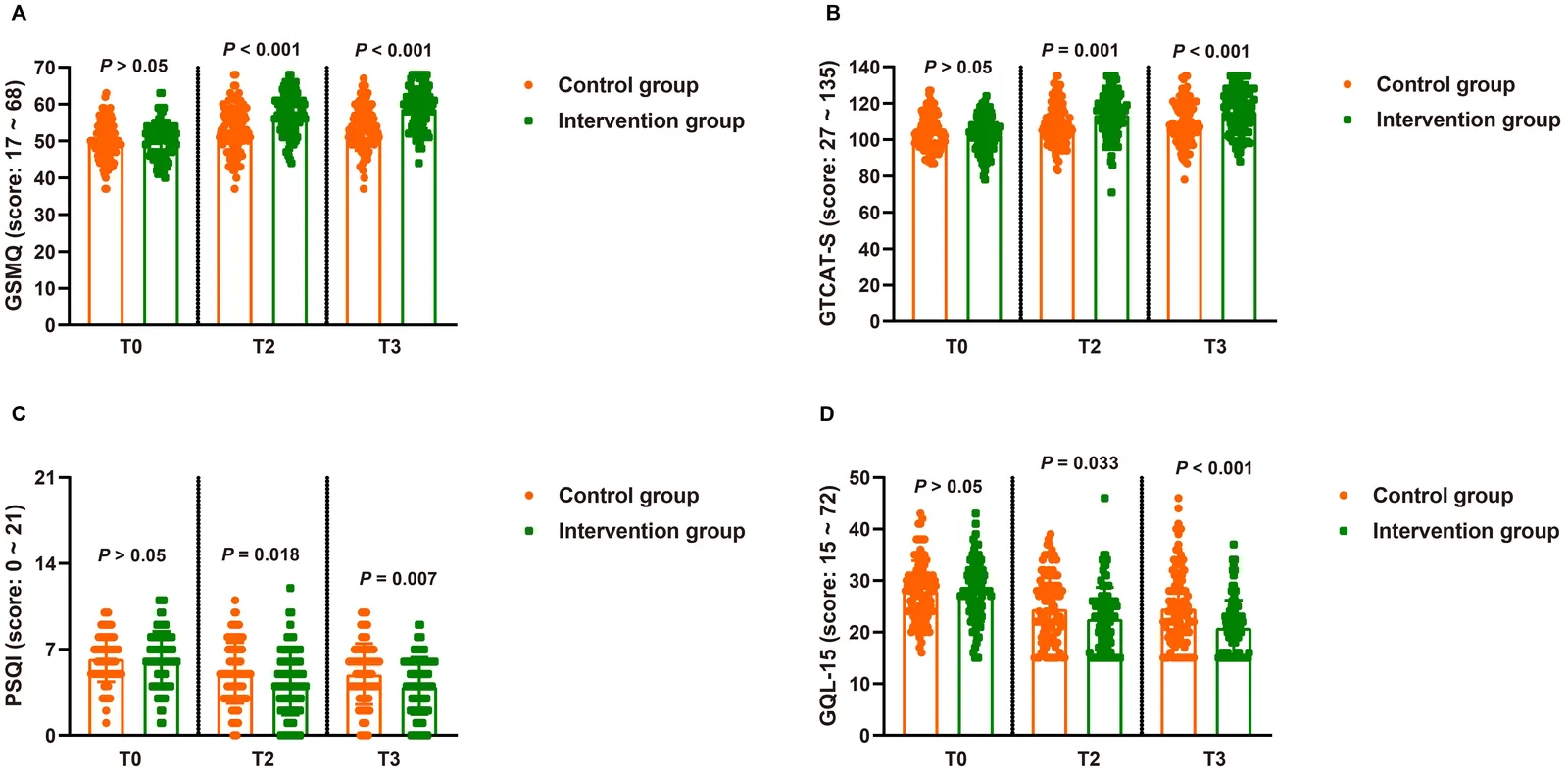
Comparisons of self-management, medication adherence, sleep quality, and vision-related quality of life between the two groups at different time points. **(A)** Glaucoma Self-Management Questionnaire (GSMQ) scores, **(B)** Glaucoma Treatment Compliance Assessment Tool-Short form (GTCAT-S) scores, **(C)** Pittsburgh Sleep Quality Index (PSQI) scores, and **(D)** Glaucoma Quality of Life-15 (GQL-15) scores in the control group and intervention group at 1 day before surgery (T0), 1 month after surgery (T2), and 3 months after surgery (T3). Values are presented as mean ± SD.

### Objective ophthalmic outcomes and major postoperative management

Repeated-measures generalized estimating equation analysis demonstrated a significant time effect for postoperative IOP (*P <* 0.001), whereas the group × time interaction was not significant (*P =* 0.868, **Table 4**). Time-specific between-group differences were estimated using linear models adjusted for baseline IOP. At postoperative day 1, mean intraocular pressure was 9.27 ± 3.18 mmHg in the control group and 8.71 ± 2.65 mmHg in the intervention group. At 3 months, the corresponding values were 12.26 ± 1.86 and 12.08 ± 2.05 mmHg. The baseline-adjusted between-group difference at 3 months was -0.19 mmHg (95% CI, -0.74 to 0.36; *P =* 0.494). No statistically significant between-group difference was detected at any postoperative intraocular-pressure assessment. Best-corrected visual acuity showed a temporary deterioration at 1 month, followed by recovery toward baseline at 3 months in both groups. Repeated-measures generalized estimating equation analysis showed no significant group × time interaction for BCVA (*P =* 0.745). Time-specific between-group differences were estimated using linear models adjusted for baseline logMAR BCVA. At 3 months, mean BCVA was 0.33 ± 0.18 logMAR in the control group and 0.29 ± 0.19 logMAR in the intervention group. The baseline-adjusted between-group difference was 0.001 logMAR (95% CI, -0.02 to 0.02; *P =* 0.941). A deterioration of at least 0.20 logMAR from baseline occurred in 4 patients (4.0%) in each group (Fisher’s exact *P =* 1.000). The median number of postoperative glaucoma medications was 0 (IQR, 0-0) in both groups at 1 and 3 months. At 3 months, 89 patients (89.9%) in the control group and 90 patients (90.9%) in the intervention group were medication-free (RR, 1.01; 95% CI, 0.93-1.10; Fisher’s exact *P =* 1.000). At least one postoperative procedure was performed in 31 patients (31.3%) in the control group and 27 patients (27.3%) in the intervention group (RR, 0.87; 95% CI, 0.56-1.34; Fisher’s exact *P =* 0.640). Major postoperative procedures were required in 5 patients (5.1%) and 3 patients (3.0%), respectively (RR, 0.60; 95% CI, 0.15-2.44; *P =* 0.721). The frequencies of individual postoperative procedures are presented in **Supplementary Table S1**. Laser suture lysis was performed in 16 patients (16.2%) in each group. Releasable suture removal was performed in 10 patients (10.1%) in the control group and 9 patients (9.1%) in the intervention group, whereas subconjunctival antifibrotic injections were administered to 15 (15.2%) and 13 (13.1%) patients, respectively. Bleb needling was performed in 7 patients (7.1%) in the control group and 6 patients (6.1%) in the intervention group. More intensive procedures were uncommon. Anterior-chamber reformation was performed in 3 patients (3.0%) and 2 patients (2.0%), wound or bleb revision in 2 patients (2.0%) and 1 patient (1.0%), and reoperation in 1 patient (1.0%) in each group. No statistically significant between-group difference was detected for any individual postoperative procedure (all Fisher’s exact *P >* 0.05).

**Table 4:** Objective ophthalmic outcomes and major postoperative management within 3 months.

Outcome	Control group (n = 99)	Intervention group (n = 99)	Effect estimate (95% CI)	*P*
Intraocular pressure, mmHg				
Postoperative day 1	9.27 ± 3.18	8.71 ± 2.65	-0.58 (-1.40 to 0.24)	0.165
Postoperative week 1	10.30 ± 4.66	9.93 ± 3.42	-0.32 (-1.47 to 0.82)	0.579
Postoperative month 1	12.71 ± 4.69	12.77 ± 4.82	0.01 (-1.32 to 1.34)	0.990
Postoperative month 3	12.26 ± 1.86	12.08 ± 2.05	-0.19 (-0.74 to 0.36)	0.494
Best-corrected visual acuity, logMAR				
Baseline	0.32 ± 0.17	0.29 ± 0.17	-0.03 (-0.08 to 0.01)	0.166
Postoperative month 1	0.39 ± 0.19	0.35 ± 0.20	-0.00 (-0.02 to 0.02)	0.868
Postoperative month 3	0.33 ± 0.18	0.29 ± 0.19	0.00 (-0.02 to 0.02)	0.941
Change from baseline to month 3	0.01 ± 0.07	0.01 ± 0.08	0.00 (-0.02 to 0.02)	0.952
Decline ≥0.20 logMAR at month 3, n (%)	4 (4.0)	4 (4.0)	RR 1.00	1.000
Postoperative glaucoma-medication burden				
Medication counts at month 1, median (IQR)	0 (0-0)	0 (0-0)	—	0.827
Medication counts at month 3, median (IQR)	0 (0-0)	0 (0-0)	—	0.816
Medication-free at month 3, n (%)	89 (89.9)	90 (90.9)	RR 1.01 (0.93-1.10)	1.000
Postoperative management				
Any postoperative procedure, n (%)	31 (31.3)	27 (27.3)	RR 0.87 (0.56-1.34)	0.640
Major postoperative procedure, n (%)	5 (5.1)	3 (3.0)	RR 0.60 (0.15-2.44)	0.721

Continuous data are presented as mean ± SD unless otherwise indicated. Repeated postoperative IOP and BCVA were analyzed using generalized estimating equations; time-specific adjusted differences represent intervention minus control and were estimated using linear models adjusted for the corresponding baseline value. Medication counts were compared using the Mann-Whitney U test. Binary outcomes were compared using Fisher’s exact test and are reported with risk ratios (RRs). Major postoperative procedures included anterior-chamber reformation, wound/bleb revision, or reoperation. BCVA, best-corrected visual acuity; IOP, intraocular pressure; IQR, interquartile range; logMAR, logarithm of the minimum angle of resolution.

### Postoperative complications within 3 months

Within 3 months after trabeculectomy, at least one postoperative complication was recorded in 33 patients (33.3%) in the control group and 27 patients (27.3%) in the intervention group. The corresponding risk ratio was 0.82 (95% CI, 0.53-1.25; *P =* 0.440, **Table 5**). At least two complication categories were recorded in 9 patients (9.1%) and 5 patients (5.1%), respectively (RR, 0.56; 95% CI, 0.19-1.60; *P =* 0.407). Transient hypotony was the most frequently recorded individual complication, occurring in 11 patients (11.1%) in the control group and 9 patients (9.1%) in the intervention group. Shallow anterior chamber occurred in 10 (10.1%) and 8 (8.1%) patients, respectively, whereas wound or bleb leakage occurred in 9 (9.1%) and 7 (7.1%) patients. Choroidal detachment or effusion, hyphema, and transient intraocular-pressure spikes were less frequent in both groups. No statistically significant between-group difference was detected for any individual complication category (all *P >* 0.05). According to the highest complication severity recorded for each patient, mild, moderate, and severe complications occurred in 20 (20.2%), 8 (8.1%), and 5 (5.1%) patients in the control group and in 17 (17.2%), 7 (7.1%), and 3 (3.0%) patients in the intervention group, respectively. The overall distribution of maximum complication severity did not differ significantly between groups (*P =* 0.338).

**Table 5:** Postoperative complications within 3 months in the control and intervention groups.

Outcome	Control group (n = 99)	Intervention group (n = 99)	Risk ratio (95% CI)	*P*
At least one complication	33 (33.3)	27 (27.3)	0.82 (0.53-1.25)	0.440
At least two complication categories	9 (9.1)	5 (5.1)	0.56 (0.19-1.60)	0.407
Transient hypotony	11 (11.1)	9 (9.1)	0.82 (0.35-1.89)	0.814
Wound/bleb leak	9 (9.1)	7 (7.1)	0.78 (0.30-2.01)	0.795
Shallow anterior chamber	10 (10.1)	8 (8.1)	0.80 (0.33-1.94)	0.806
Choroidal detachment/effusion	5 (5.1)	4 (4.0)	0.80 (0.22-2.89)	1.000
Hyphema	5 (5.1)	3 (3.0)	0.60 (0.15-2.44)	0.721
Transient IOP elevation/spike	7 (7.1)	5 (5.1)	0.71 (0.23-2.17)	0.767
Maximum complication severity			—	0.338
Mild	20 (20.2)	17 (17.2)	0.85 (0.47-1.52)	0.716
Moderate	8 (8.1)	7 (7.1)	0.87 (0.33-2.32)	1.000
Severe	5 (5.1)	3 (3.0)	0.60 (0.15-2.44)	0.721

Data are presented as n (%). A patient could experience more than one complication and was counted in each applicable category; composite outcomes counted each patient once. Mild events required observation or topical treatment only; moderate events required additional medication or an office-based procedure; severe events required anterior-chamber reformation, wound/bleb revision, or reoperation. Risk ratios compare the intervention group with the control group. Binary outcomes and individual severity categories were compared using two-sided Fisher’s exact tests; the overall distribution of maximum complication severity was compared using the Mann-Whitney U test. IOP, intraocular pressure.

## Discussion

The present study showed that perioperative nursing based on Mishel’s Uncertainty in Illness Theory was associated with reduced illness uncertainty, anxiety, and depression, as well as improved discharge readiness, satisfaction with nursing care, self-management behavior, medication adherence, sleep quality, and vision-related quality of life in patients with primary glaucoma undergoing trabeculectomy. According to Mishel’s theory, illness uncertainty occurs when patients are unable to assign clear meaning to illness-related events because symptoms, treatment information, prognosis, or recovery processes are ambiguous or difficult to predict (10, 15). From this perspective, the present intervention was not merely a conventional health education program, but a structured nursing strategy aimed at improving patients’ interpretation of disease- and surgery-related information, strengthening coping resources, and promoting postoperative adaptation. However, the intervention did not significantly reduce postoperative complications within 3 months, suggesting that its main benefits were psychological, behavioral, and recovery-related rather than directly surgical.

A key finding of this study was the greater reduction in illness uncertainty in the intervention group. Glaucoma is a chronic, progressive, and potentially blinding disease with complex treatment requirements and an uncertain visual prognosis (1), which may heighten uncertainty regarding disease progression, surgical outcomes, recovery, and long-term control. Previous studies have similarly linked illness uncertainty in glaucoma with anxiety and depressive symptoms (8, 9). In the present study, individualized education, repeated explanation, teach-back, perioperative guidance, and family involvement may have reduced ambiguity by helping patients understand the purpose of trabeculectomy, expected postoperative changes, warning symptoms, medication requirements, and follow-up needs. The individualized approach is also consistent with evidence that psychological and interpersonal needs vary substantially between patients (34) and with a systematic review identifying informational support as a core component of uncertainty-management interventions (35).

The accompanying reductions in anxiety and depression suggest that clearer illness representations may also support emotional adjustment. At T3, the model-estimated MUIS difference was -8.13 points, whereas HADS-A and HADS-D differences were smaller (-1.17 and -1.71 points); lower proportions above the HADS cutoff of 8 support possible clinical relevance, although these secondary effects remain exploratory. Emotional adjustment may depend not only on the amount of information provided but also on how patients appraise affective experiences and communicate concerns. Evidence from other psychiatric populations supports the broader psychosomatic relevance of emotional appraisal and relational support (36), consistent with Mishel’s framework in which appraisal shapes emotional responses and coping adaptation (11, 15). Similar uncertainty-management interventions in cancer populations have also improved knowledge, communication, coping skills, and psychological adjustment (37, 38).

The intervention group also showed greater discharge readiness and satisfaction with nursing care at discharge. This finding is important because discharge after trabeculectomy represents a critical transition from hospital-based care to home-based self-care. Patients must use postoperative eye drops correctly, protect the filtering bleb, avoid inappropriate activities, recognize warning signs, and attend scheduled follow-up visits. The structured intervention clarified postoperative expectations, reinforced key self-care information, and encouraged family participation, which may have enhanced confidence and perceived preparedness. Higher satisfaction with nursing care may also reflect that patients perceived the intervention as more responsive to their informational and emotional needs. More broadly, multidisciplinary psychosomatic care models conceptualize nursing as a therapeutic function integrating continuity, structured monitoring, emotional support, and patient-centered communication (39).

The favorable between-group differences in self-management behavior and medication adherence suggest sustained behavioral benefits during follow-up. Although trabeculectomy can reduce IOP and medication burden, postoperative recovery and long-term glaucoma control still depend on patient engagement. Educational interventions may improve glaucoma medication adherence when they address knowledge, beliefs, and practical barriers (40), and nurse-led education may strengthen health literacy and self-management activation (23). In the present study, teach-back and repeated guidance may have helped patients translate perioperative information into practical self-care behaviors, thereby strengthening perceived control and postoperative adaptation.

The intervention group also showed better sleep quality and vision-related quality of life during follow-up. These outcomes are likely multifactorial: reduced psychological distress may support sleep, while clearer understanding of postoperative care may reduce worry and strengthen confidence. The observed differences may therefore reflect reduced uncertainty, improved emotional adjustment, and enhanced self-management capacity. Because no statistically significant between-group differences were detected in postoperative IOP trajectories, BCVA recovery, medication-free status, or major postoperative procedures, the GQL-15 and self-management findings were unlikely to be explained solely by short-term surgical outcomes. This interpretation is consistent with qualitative recovery literature emphasizing symptom change, meaning-making, interpersonal support, and perceived agency (41). Nevertheless, nonspecific attention, expectation, satisfaction, and Hawthorne effects remain plausible because the intervention group received more structured contact than the routine-care group.

No statistically significant between-group difference was detected in postoperative complications. Early events after trabeculectomy, including hyphema, hypotony, bleb leakage, shallow anterior chamber, choroidal detachment, and IOP fluctuation, are influenced mainly by surgical technique, wound healing, ocular tissue response, and individual anatomy or disease characteristics (42, 43). Structured nursing care may improve symptom recognition, timely reporting, adherence, and follow-up, but it may not substantially alter the biological occurrence of surgery-related complications during short follow-up. Moreover, the sample size was calculated for MUIS rather than relatively infrequent safety events; therefore, the nonsignificant findings should not be interpreted as evidence of equivalent complication rates.

## Limitations

Several limitations should be acknowledged. First, this single-center study with a 3-month follow-up may limit the generalizability of the findings and preclude assessment of the long-term sustainability of the intervention effects. Second, most outcomes were self-reported and may have been affected by recall, reporting, and social-desirability bias. Third, participants and intervention nurses could not be blinded, and outcome assessors and statistical analysts were not formally blinded; therefore, performance, detection, and analytical bias cannot be excluded. Fourth, the control condition was not attention-matched, and the additional nurse contact, reassurance, and telephone follow-up received by the intervention group may have contributed to the observed effects. Fifth, although the intervention followed a structured protocol, fidelity was not independently audited, and the duration of contacts, patient participation, protocol deviations, and family involvement were not prospectively recorded, limiting assessment of intervention dose and reproducibility. Finally, despite Holm adjustment, the multiple secondary outcomes and ophthalmic analyses should be considered exploratory. Filtering-bleb morphology, visual-field progression, and objectively measured medication adherence were not evaluated. Future multicenter studies with longer follow-up should incorporate repeated patient-reported outcome assessment and prespecified subgroup analyses to identify potentially heterogeneous recovery trajectories and treatment effects (44, 45).

## Conclusions

In this single-center randomized study, perioperative nursing based on Mishel’s Uncertainty in Illness Theory was associated with greater improvements in illness uncertainty, emotional symptoms, discharge readiness, self-management, medication adherence, sleep quality, and vision-related quality of life during the first 3 months after trabeculectomy. These findings support the potential value of structured uncertainty-focused nursing as an adjunct to routine ophthalmic care, while the modest magnitude of some secondary effects and the risk of nonspecific attention and self-report bias should be considered. No statistically significant reduction in postoperative complications was detected.

## Data Availability

The original contributions presented in the study are included in the article/**Supplementary Material**. Further inquiries can be directed to the corresponding author.
