# Supplementary material for: Mishel theory-based perioperative nursing after trabeculectomy for primary glaucoma: effects on illness uncertainty, self-management, and quality of life
Source: Front Psychiatry. 2026 Oct 14;17:1884293. doi: 10.3389/fpsyt.2026.1884293 (PMC13713672; doi:10.3389/fpsyt.2026.1884293)
Supplement: Supplementary file 1 [file Table1.docx]

**Supplementary Table S1. Detailed postoperative procedures within 3 months**

| Procedure | Control group (n = 99) | Intervention group (n = 99) | Risk ratio (95% CI) | *P* |
| --- | --- | --- | --- | --- |
| Laser suture lysis | 16 (16.2) | 16 (16.2) | 1.00 (0.53-1.89) | 1.000 |
| Releasable suture removal | 10 (10.1) | 9 (9.1) | 0.90 (0.38-2.12) | 1.000 |
| Subconjunctival antifibrotic injection | 15 (15.2) | 13 (13.1) | 0.87 (0.44-1.73) | 0.839 |
| Bleb needling | 7 (7.1) | 6 (6.1) | 0.86 (0.30-2.46) | 1.000 |
| Anterior chamber reformation | 3 (3.0) | 2 (2.0) | 0.67 (0.11-3.90) | 1.000 |
| Wound/bleb revision | 2 (2.0) | 1 (1.0) | 0.50 (0.05-5.43) | 1.000 |
| Reoperation | 1 (1.0) | 1 (1.0) | 1.00 (0.06-15.77) | 1.000 |

Notes: Data are presented as n (%). A patient could receive more than one postoperative procedure and was counted in each applicable category. Risk ratios compare the intervention group with the control group. *P* values were calculated using two-sided Fisher’s exact tests.
