# Supplementary material for: Mishel theory-based perioperative nursing after trabeculectomy for primary glaucoma: effects on illness uncertainty, self-management, and quality of life
Source: Front Psychiatry. 2026 Oct 14;17:1884293. doi: 10.3389/fpsyt.2026.1884293 (PMC13713672; doi:10.3389/fpsyt.2026.1884293)
Supplement: Supplementary file 2 [file Table2.docx]

**Supplementary Table S2. Psychometric properties of the patient-reported outcome measures used in this study**

| **Instrument** | **Construct and structure** | **Scoring and interpretation** | **Psychometric properties** | **Reference** |
| --- | --- | --- | --- | --- |
| MUIS | 28 items covering ambiguity, complexity, inconsistency, and unpredictability | 5-point Likert scale; total score 28–140; higher scores indicate greater illness uncertainty | The MUIS has demonstrated adequate-to-excellent reliability and good validity, including previous use and validation in Chinese ophthalmic populations. | Mishel 1981; Zhang et al. 2018 |
| HADS | 14 items comprising two 7-item subscales: HADS-A and HADS-D | Each item scored 0–3; each subscale ranges from 0–21; higher scores indicate greater symptom severity | Chinese versions have demonstrated good internal consistency, construct validity, and concurrent validity. Reported Cronbach’s α values for HADS-A and HADS-D were approximately 0.75 and 0.76, respectively, in Chinese clinical populations. | Fomenko et al. 2025 |
| RHDS | 12 items across three dimensions: personal status, coping ability, and expected support | Each item scored 0–10; mean item score 0–10; higher scores indicate greater discharge readiness | The Chinese version has demonstrated good internal consistency and construct validity; previous studies report Cronbach’s α of approximately 0.89 for the overall scale. | Lin et al. 2014 |
| GSMQ | 17 items across three dimensions: life adjustment, functional health care, and medical management | Four response categories scored 1–4; total score 17–68; higher scores indicate better self-management | The Chinese version demonstrated good reliability and validity, with Cronbach’s α coefficients across dimensions ranging from 0.712 to 0.891. | Zhang et al. 2025 |
| GTCAT-S | 27 statements reflecting five Health Belief Model components: knowledge, susceptibility, cues to action, self-efficacy, and barriers | 5-point Likert responses; higher scores reflect more favorable adherence-related beliefs and behaviors | Principal-component analysis supported a five-component structure, and the 27-item version showed predictive validity for objectively measured adherence (R² = 0.11, P = 0.03). | Barker and Mansberger 2019 |
| PSQI | 19 self-rated items generating seven component scores | Global score 0–21; higher scores indicate poorer sleep quality | The PSQI has demonstrated good internal consistency and test–retest reliability; Chinese versions have also shown acceptable reliability and construct validity. | Buysse et al. 1989 |
| GQL-15 | 15-item glaucoma-specific vision-related quality-of-life measure | Higher scores indicate poorer vision-related quality of life | The Chinese version demonstrated excellent internal consistency (Cronbach’s α = 0.96), with subscale α values of 0.75–0.91 and test–retest ICCs >0.70. | Zhou et al. 2013 |
| NSNS | 19-item satisfaction subscale assessing patients’ satisfaction with nursing care | Scores converted to a 0–100 scale; higher scores indicate greater satisfaction with nursing care | The original scale demonstrated satisfactory construct validity and internal consistency and was able to discriminate between hospitals and wards. | Thomas et al. 1996 |

Notes: MUIS, Mishel Uncertainty in Illness Scale; HADS, Hospital Anxiety and Depression Scale; HADS-D, Hospital Anxiety and Depression Scale–Depression; RHDS-SF, Readiness for Hospital Discharge Scale–Short Form; GSMQ, Glaucoma Self-Management Questionnaire; GTCAT-S, Glaucoma Treatment Compliance Assessment Tool–Short Form; PSQI, Pittsburgh Sleep Quality Index; GQL-15, Glaucoma Quality of Life-15; NSNS, Newcastle Satisfaction with Nursing Scales.
